# Aptamers Against the β-Conglutin Allergen: Insights into the Behavior of the Shortest Multimeric(Intra)Molecular DNA G-Quadruplex

**DOI:** 10.3390/ijms22031150

**Published:** 2021-01-24

**Authors:** Ciara K. O’ Sullivan, Teresa Mairal, Miriam Jauset-Rubio, Marketa Svobodova, Vasso Skouridou, Veronica Esposito, Antonella Virgilio, Aldo Galeone

**Affiliations:** 1 INTERFIBIO Research Group, Departament d’Enginyeria Química, Universitat Rovira i Virgili, Avinguda Països Catalans 26, 43007 Tarragona, Spain; teresa.mairal@urv.cat (T.M.); miriam.jauset@urv.cat (M.J.-R.); marketa.svobodova@urv.cat (M.S.); vasoula.skouridou@urv.cat (V.S.); 2 Institució Catalana de Recerca i Estudis Avançats (ICREA), Passeig Lluís Companys 23, 08010 Barcelona, Spain; 3 Dipartimento di Farmacia, Università degli Studi di Napoli Federico II, Via D. Montesano 49, 80131 Napoli, Italy; verespos@unina.it (V.E.); antonella.virgilio@unina.it (A.V.); galeone@unina.it (A.G.)

**Keywords:** G-quadruplex, 11-mer aptamer, β-conglutin, PAGE, melting curves, CD, NMR, BLI, MST, SPR

## Abstract

In previous work, a 93-mer aptamer was selected against the anaphylactic allergen, β-conglutin and truncated to an 11-mer, improving the affinity by two orders of magnitude, whilst maintaining the specificity. This 11-mer was observed to fold in a G-quadruplex, and preliminary results indicated the existence of a combination of monomeric and higher-order structures. Building on this previous work, in the current study, we aimed to elucidate a deeper understanding of the structural forms of this 11-mer and the effect of the structure on its binding ability. A battery of techniques including polyacrylamide gel electrophoresis, high-performance liquid chromatography in combination with electrospray ionization time-of-flight mass spectrometry, matrix-assisted laser desorption/ionization time-of-flight, thermal binding analysis, circular dichroism and nuclear magnetic resonance were used to probe the structure of both the 11-mer and the 11-mer flanked with TT- at either the 5′ or 3′ end or at both ends. The TT-tail at the 5′ end hinders stacking effects and effectively enforces the 11-mer to maintain a monomeric form. The 11-mer and the TT- derivatives of the 11-mer were also evaluated for their ability to bind its cognate target using microscale thermophoresis and surface plasmon resonance, and biolayer interferometry confirmed the nanomolar affinity of the 11-mer. All the techniques utilized confirmed that the 11-mer was found to exist in a combination of monomeric and higher-order structures, and that independent of the structural form present, nanomolar affinity was observed.

## 1. Introduction

Aptamers are single-stranded DNA or RNA oligonucleotides capable of binding their cognate target with high affinity and specificity. Targets for aptamer selection range from small molecules [1] and proteins [2] to very complex target types, such as whole cells [3], viruses [4] or even whole tissues [5]. Aptamers are generated through a combinatorial technique called Systematic Evolution of Ligands by Exponential Enrichment (SELEX) [6,7,8], in which a specific sequence is selected by iterative rounds of systematic binding, competition, selection, amplification and enrichment. Compared to antibodies, aptamers offer several unambiguous advantages due to their smaller size and nucleic acid characteristics that improve their applicability. The investigation of specific structural features of aptamers might help to understand their behavior and their binding abilities, particularly in view of suitable post-SELEX modifications required for many applications [9].

Owing to a range of intramolecular interactions, aptamers fold into well-defined three-dimensional structures, often endowed with a remarkable thermal stability and an appropriate flexibility, essential for the interaction with the target. Many aptamers have the ability to fold into stable G-quadruplex structures under physiological conditions, and these aptamers have several advantages compared with unstructured sequences. They are thermodynamically and chemically stable, show no immunogenicity and are resistant to numerous serum nucleases [10]. The structural unit of a G-quadruplex is a G-tetrad composed of four guanine residues held together by Hoogsteen hydrogen bonds in a planar ring configuration [11,12]. The G-quadruplex contains two or more G-tetrads, and based on the relative strand directions, they can be classified into parallel, antiparallel or mixed topologies [13]. Highly complexed G-quadruplex structures are frequently located in human promoter regions, in telomeres [14] and 5′ untranslated regions of mRNAs [15]. The RNA G-quadruplexes generally adopt parallel G-quadruplex structures [16], whilst DNA G-quadruplex counterparts display a higher diversity in strand orientation and can adopt antiparallel [17], parallel [18] and hybrid conformations [19], depending on many factors, including the type of loops and cations, the nucleotide number in the G-runs and the main strand polarity of the G-quadruplex [20]. The presence of G-quadruplex aptamers and their folding/unfolding processes play important roles in many biological functions and became interesting in a therapeutic diagnosis [10,21]. 

In recent years, aptamers have been increasingly employed for food safety monitoring [22,23], and an emerging area of application is the detection of food allergens. In 2012, we reported the selection of a high affinity aptamer against β-conglutin [24], a lupine protein that has been identified by the International Union of Immunological Societies as the Lup an 1 allergen. The original selected β-conglutin binding aptamer (βCBA) was truncated from a 93-mer to a 11-mer [25], improving its affinity by two orders of magnitude, and to the best of our knowledge, to date, this 11-mer is the shortest G-quadruplex aptamer with nanomolar affinity. The 11-mer was successfully exploited in a FRET-based probe, and the results obtained indicated that this highly sensitive and specific truncated aptamer had a complex structure, and a further understanding of this structure was subsequently explored [26]. Preliminary nuclear magnetic resonance (NMR) results strongly suggested that the 11-mer adopts a G-quadruplex structure as expected by considering its G-rich sequence similar to the thrombin binding aptamer (TBA) [27]. However, compared to most of the other G-rich sequences able to adopt a G-quadruplex structure, the 11-mer shows peculiar features due to the high percentage of G residues and their presence at both the 3′ and 5′ ends. Indeed, these characteristics often promote a structural polymorphism and/or the formation of high-order structures. 

In this study, a thorough and systematic investigation was carried out in order to elucidate the peculiar architectures adopted by 11-mer and provide insight into its mechanism of action. A battery of biophysical techniques was employed, including gel electrophoresis, circular dichroism, mass spectrometry and NMR spectroscopy, providing an understanding of the folding topology of G-quadruplex. In parallel, the binding affinities of the 11-mer and related sequences were determined using a wide range of techniques used for biomolecular interactions, including surface plasmon resonance (SPR), bio-layer interferometry (BLI) and microscale thermophoresis (MST).

## 2. Results

### 2.1. Structural Studies

#### 2.1.1. Polyacrylamide Gel Electrophoresis (PAGE)

**Figure 1:**
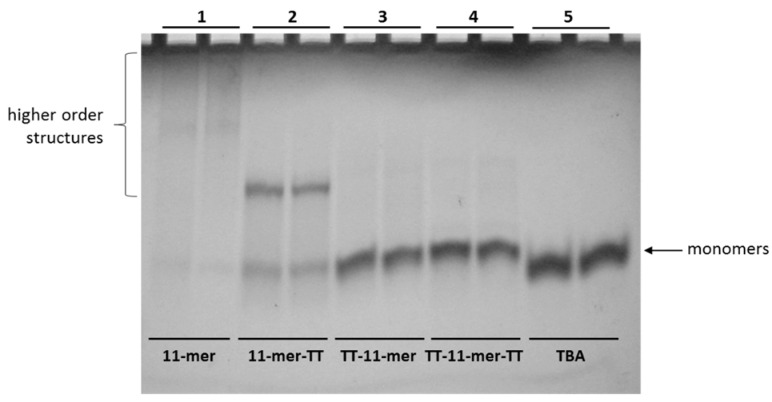
Polyacrylamide gel electrophoresis analysis of 11-mer and its derivatives. Lane 1: 11-mer; lane 2: 11-mer-TT; lane 3: TT-11-mer; lane 4: TT-11-mer-TT; lane 5: TBA. Each oligonucleotide was analyzed in duplicate.

As reported by other authors, a helpful approach to avoid the formation of higher-order structures and reduce the G-quadruplex polymorphism consists of extending the original sequence with further residues at the 3′ and/or 5′ end. Additionally, sequences with guanines in their 5′ end favor the stacking of G-quadraplexes, and as a result, dimers and higher-order structures can be formed [28]. In several cases, the addition of one or more thymine residues at the sequence ends has proven to be particularly successful in avoiding the formation of these higher-order structures [29]. With these results in view, we designed three 11-mer-related sequences in which a TT tail was added to the 3′ or/and the 5′ end of the original sequence (Table S1) and analyzed them by polyacrylamide gel electrophoresis. In Figure 1, the non-denaturing gel profile obtained in the presence of sodium ions is shown. The presence of an extended smeared band in the case of the parent 11-mer has strongly confirmed the propensity of this sequence to form higher-order G-quadruplex structures. On the other hand, the electrophoretic behavior of its TT-tailed-related sequences appears quite different. Migrations of all the extended sequences are characterized by the significant absence of the smeared band, indicative of higher-order structures. Furthermore, all the TT-tailed sequences show very similar bands whose migration is comparable to that of the two-tetrads monomolecular G-quadruplex formed by the thrombin binding aptamer (TBA) used as a reference [27], thus suggesting the formation of monomolecular structures in these conditions also for the 11-mer-related sequences. However, 11-mer-TT shows a further slower distinct migrating band, suggestive of the formation of a G-quadruplex structure involving more than one oligonucleotide molecule. In summary, the electrophoretic investigations clearly indicate that the presence of one or two TT-tails has been able to decrease the tendency of the original 11-mer to form higher-order structures. However, the electrophoretic behavior of 11-mer-TT would suggest a higher propensity of the free 5′ end to induce polymorphism and formation of a high-order structure, compared to the 3′ end.

#### 2.1.2. Liquid Chromatography Coupled with Electrospray Ionization Time-of-Flight Mass Spectrometry (HPLC-ESI-TOF-MS)

**Figure 2:**
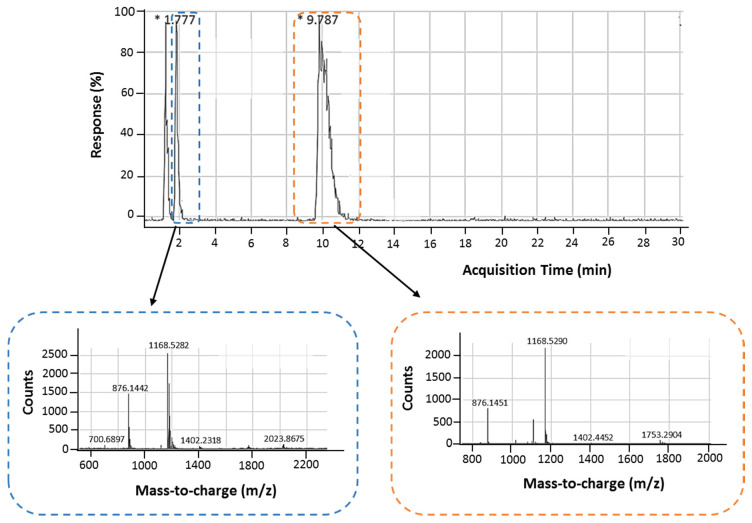
HPLC-ESI-TOF-MS analysis of the 11-mer. The top panel is the chromatogram showing the HLPC separation of the monomeric and multimeric structures, and the bottom panels show the MS for each peak obtained in HPLC.

High-performance liquid chromatography (HPLC) was performed in combination with electrospray ionization time-of-flight mass spectrometry (HPLC-ESI-TOF-MS). As seen in the HPLC chromatogram (Figure 2), two different retention times were obtained: the first at 1.78 min was assigned to monomeric 11-mer aptamer and the second at 9.79 min to higher-order structures, and these results were in agreement with gel electrophoresis. Following ESI-TOF-MS, the mass-to-charge is observed to be 1168.5282 and 1168.5290 (the m/z value of 1168 matches z = 3), corresponding to the peaks at 1.78 and 9.79 min, respectively. This clearly demonstrates that the second peak observed in HPLC is indeed a higher-ordered structure of the monomer, but we cannot elucidate the exact form of this structure. 

The 11-mer and its TT derivatives were also analyzed by matrix-assisted laser desorption/ionization time-of-flight (MALDI-TOF). The peak corresponding to higher-order structures observed for the 11-mer (mass-to-charge of 7106.671) is not present in the spectra of the TT derivatives, thus confirming the ability of the TT extensions to prevent stacking and maintain the monomeric form (Figure S1).

#### 2.1.3. Thermal Melting Analysis

**Figure 3:**
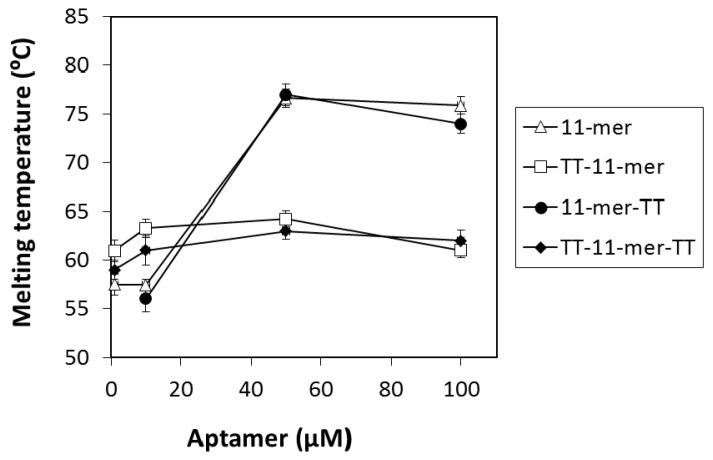
UV melting analysis of 11-mer and its TT derivatives at different oligonucleotide concentrations (1–100 μM). Error bars represent the average of duplicate experiments.

Thermal melting analysis is another useful technique used to elucidate information regarding the structure of DNA. The melting temperatures of the 11-mer, as well as its derivatives with TT extensions, were measured at 295 nm at different concentrations of the oligonucleotides (Figure 3).The melting assays were carried out in binding buffer supplemented with 20 mM KCl, as the presence of this salt increases the stability of G-quadruplex structures [30]. The UV melting temperatures observed for the 11-mer with the TT extensions at the 5′ end (TT-11-mer and TT-11-mer-TT) appeared to be independent of the oligonucleotide concentration suggesting monomer states and the avoidance of stacking. Concentration-dependent melting curves, however, were observed for the unmodified 11-mer and its derivative with the TT extension at the 3′ end, suggesting multimeric G-quadruplex formation. Similar effects have been previously reported for the 93del dimeric parallel G-quadruplex [31]. The melting temperatures (Tm) obtained for the 100 μM oligonucleotide solutions can be found in Table S2 and Figure S2. The Tm of the 11-mer was observed to be 76 °C, and when the TT extension was added to the 3′ end (11-mer-TT), the Tm was similar (75 °C). However, when the TT extensions were added to the 5′ end, the Tm was significantly reduced to 61 and 62 °C, for the TT-11-mer and TT-11-mer-TT, respectively. This re-affirms the results obtained using gel electrophoresis, again suggesting that the 11-mer exists in a mixture of monomeric and dimeric/multimeric forms, where the introduction of the TT extension at the 5′ end prevents stacking [31] and, thus, prevents the formation of higher-order structures. These results again clearly indicate that the 11-mer is not solely a monomer and exists as a mixture of monomer and higher-order structures.

#### 2.1.4. Circular Dichroism (CD)

**Figure 4:**
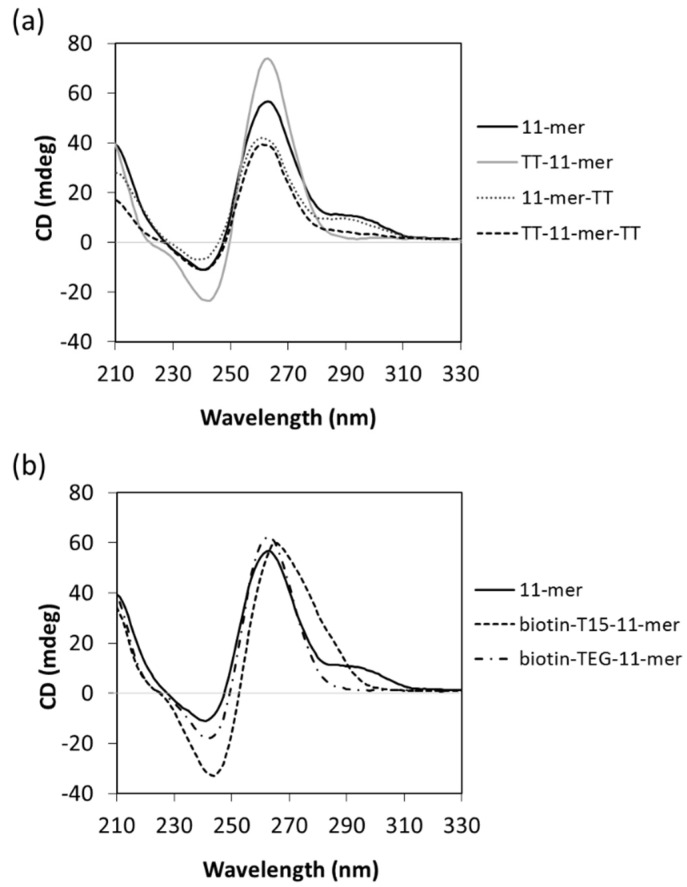
CD spectra of (**a**) the 11-mer and TT derivatives and (**b**) biotinylated derivatives. The spectra were obtained using 10 µM of each oligonucleotide and represent the average of three measurements.

The interactions of guanosines are different in parallel and anti-parallel G-quadruplexes, and circular dichroism was further used to study the orientation of the 11-mer dimeric/multimeric structure [32]. As can be seen in Figure 4a and Figure S3a, for both the 11-mer and 11-mer-TT spectra, a CD negative peak is observed at 240 nm, together with a positive peak at 263 nm and a shoulder at 295 nm. This profile strongly suggests the presence of a major parallel G-quadruplex, characterized by the negative peak at 240 nm together the positive one at 263 nm, whilst the positive peak at 295 nm is suggestive of the occurrence of minor non-parallel G-quadruplexes [33,34]. When the thymine extensions were added to the 5′ end (TT-11mer or TT-11mer-TT, i.e., monomeric form of 11-mer due to prevention of stacking), the positive peak at 295 nm disappeared, indicative of a purely parallel orientation (Figure 4a).

Biotinylated aptamers are frequently used in aptamer-based assays/aptasensors, either to immobilize the aptamer or to bind to a reporter molecule. These biotinylated aptamers typically contain “spacer” molecules to give the aptamer more flexibility to fold and bind and, in the case of immobilized aptamers, to propel it from the surface to facilitate enhanced accessibility for binding. It was, thus, of interest to probe the effect of biotinylation and different spacers on the 11-mer structure. Two different modifications were used, 11-mer with a 15× thymine spacer (T15) at its 5′ end (biotin-T15-11-mer) and the 11-mer with a triethyleneglycol spacer (biotin-TEG-11-mer). As shown in Figure 4b, the CD spectrum of the biotin-TEG-11-mer has a negative peak at 240 nm, and a positive peak at 263 nm, whilst the positive peak at 295 nm is absent, indicating only parallel G-quadruplex conformation, typical of the monomer. Although the biotinylated 11-mer with a T15 spacer (biotin-T15-11-mer) shows a broad peak in the 245–300 nm area, also its CD profile is characterized by the negative and positive peaks typical of a parallel G-quadruplex conformation. These results show that when both the spacers are used at the 5′ end in order to introduce the biotin modification, the aptamer is driven to adopt the parallel monomeric conformation, in agreement with the results obtained with the modified 11-mer sequences containing 5′-TT tails. Interestingly, from an application point of view, these results suggest that using the spacers maintains the 11-mer in its monomeric form.

#### 2.1.5. Nuclear Magnetic Resonance (NMR)

**Figure 5:**
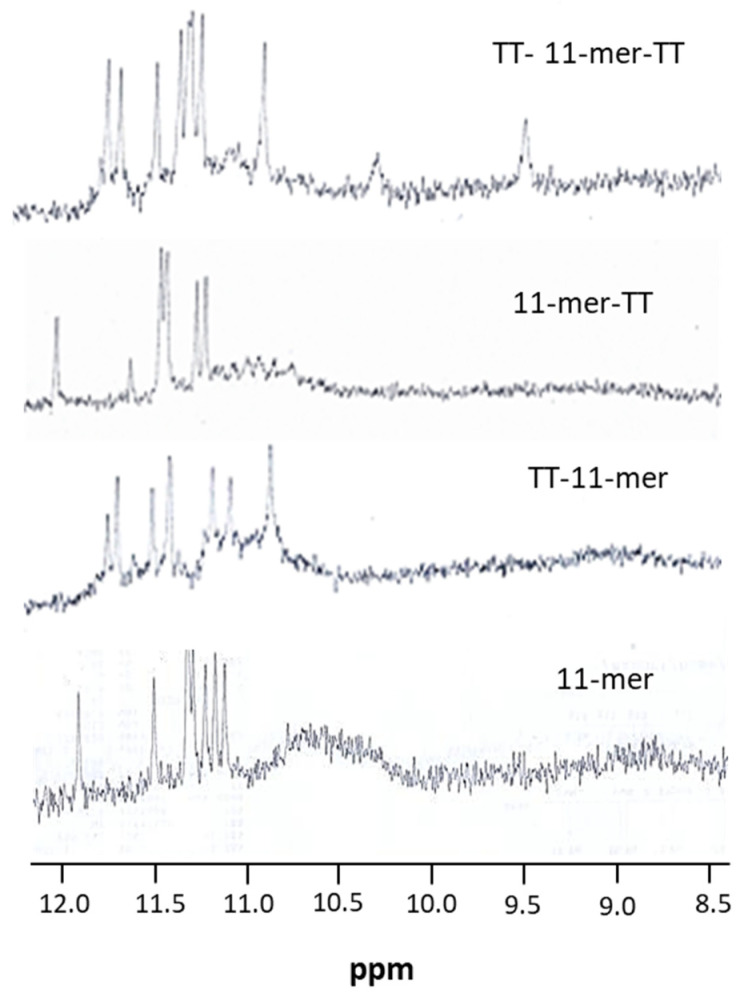
Imino and amino regions of the^1^H NMR (500 MHz) spectra of the 11-mer and its derivatives containing flanking TT at the 3′ end, 5′ end or both. See the main text and the experimental section for details.

In order to further assess the presence of G-quadruplex structures and whether the oligonucleotides adopt a unique and/or multiple conformations, a preliminary 1H-NMR analysis was performed, considering that one of the distinctive features of G-quadruplexes is the appearance of imino proton resonances in the region between 10.5 and 12.0 ppm in 1H-NMR spectra. As expected, the one-dimensional 1H-NMR spectra of the 11-mer and its TT-derivatives samples showed the presence of eight signals in the imino region (11.0–12 ppm) attributable to imino protons involved in Hoogsteen hydrogen bonds of G-quartets (Figure 5). Nevertheless, with regards to the 11-mer and 11-mer-TT, the presence of further broad signals in the range 10.5–11 ppm suggests the co-existence of minor amounts of other G-quadruplexes, high-order structures or both, in agreement with the presence of a shoulder around 295 nm in their CD profiles. Unfortunately, the high oligonucleotide concentrations required for NMR investigations, strongly promoting intermolecular aggregation, and the wide symmetry of the sequences analyzed, generating signals overlapping, prevented us from performing further NMR investigations due to the suboptimal quality of the spectra obtained. 

### 2.2. Binding Studies

**Figure 6:**
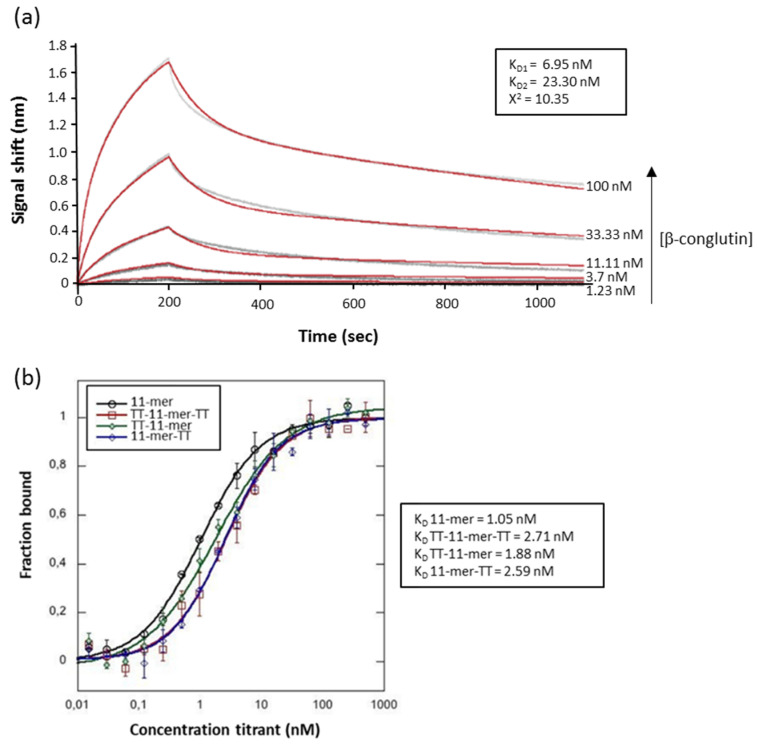
Binding studies with the 11-mer and TT derivatives. (**a**) Biolayer interferometry (BLI) and (**b**) microscale thermophoresis (MST). The concentrations of target used are included in the figure.

In order to calculate the affinity dissociation constant (K_D_) of the 11-mer, two approaches were used, with the aptamer either immobilized (biolayer interferometry (BLI)) or in solution (microscale thermophoresis (MST)). BLI is based on the real-time measurement of molecular interactions by detecting changes in interference patterns of white light reflected from the surface of fiber-optic sensor tips. In this approach, the 11-mer aptamer with a biotin-TEG-T15 modification on its 5′ end was immobilized on high-sensitivity streptavidin-activated biosensors (SAX), and following immobilization, a range of concentrations of β-conglutin were passed through the chip (Figure 6a). A 2:1 heterogenous model was used to fit the data and calculate the binding affinities resulting in two different K_D_ values of 6.95 and 23.30 nM. A 1:1 binding model was also used, but the goodness of the fit was not acceptable resulting in a poor X^2^ value (Figure S4). This could be attributed to the multimeric nature of the target β-conglutin (trimer of approximately 186 kDa), which could allow the simultaneous binding of more than one aptamer molecules.

In MST, the 11-mer was modified in the 5′ end with a Cy5 fluorophore, and the change in fluorescence upon target binding was monitored. A constant amount of modified aptamer was incubated with a range of concentrations of β-conglutin, and their interactions were monitored. Using MST, where the aptamer and target are both in solution, the affinity constants of the TT-tethered 11-mers were also analyzed, and very interestingly, despite the fact that the TT-11-mer and TT-11-mer-TT seem to be monomers (due to the prevention of stacking), whilst the 11-mer and 11-mer-TT appear to have higher-order structures, the K_D_ values determined (Figure 6b) are very similar (11-mer: 1.05 nM, TT-11-mer-TT: 2.71 nM, TT-11 mer: 1.88 nM and 11-mer-TT: 2.59 nM). It should be noted that both the BLI and MST were carried out by 2Bind GmBH and are, thus, independent evaluations of the affinity of the 11-mer and the thymine-tethered 11-mer.

**Figure 7:**
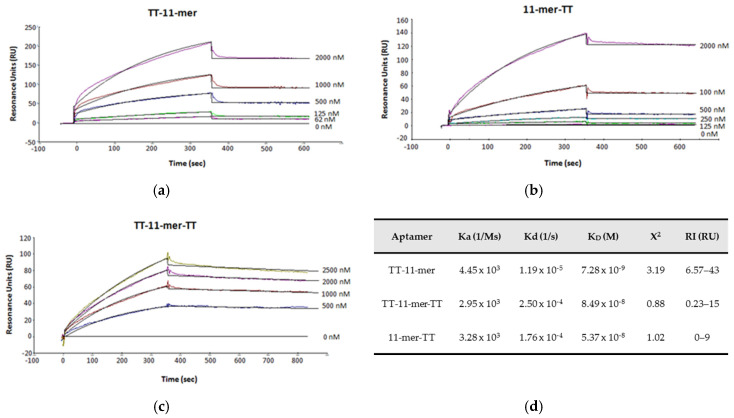
Binding studies with the 11-mer TT derivatives by surface plasmon resonance (SPR). (**a**) TT-11-mer, (**b**) 11-mer TT, (**c**) TT-11-mer-TT. (**d**) Kinetic constants used for K_D_ calculation.

Surface plasmon resonance (SPR) was also carried out to determine the affinity constants, where the β-conglutin was immobilized and exposed to a range of concentrations of the 11-mer and each of the thymine-tethered 11-mers, and again, similar affinity constants (TT-11 mer: 7.28 nM, 11-mer-TT: 84.9 nM and TT-11-mer-TT: 53.7 nM) in the low nanomolar range were obtained (Figure 7). The affinity constants were calculated using the kinetic constants and the 1:1 model, and excellent fittings were achieved as evidenced by the low X^2^ values.

## 3. Discussion

In previous studies, we selected a 93-mer aptamer against the anaphylactic food allergen β-conglutin, found in lupin. The selected aptamer had a K_D_ in the submicromolar range, and we carried out truncation studies to see if an improvement in affinity could be achieved, and in these studies, we elucidated an 11-mer with nanomolar affinity. Subsequently, we used this 11-mer in a FRET assay and obtained interesting results suggesting the presence of dimers, or even higher-order structures. This 11-mer was observed to be high in G content, and to probe the potential presence of G-quadruplex structures, a preliminary 1H-NMR analysis was performed, probing the region between 10.5 and 12.0 ppm where the imino proton resonances, distinctive features of G-quadruplex structures, occur [35,36,37]. 1H-NMR analysis can also provide information as to whether the oligonucleotide adopts a unique structure and to give insight into its symmetry. In these preliminary studies, the quality of the spectra was far from optimal, but the peak pattern clearly indicated the co-existence of a major G-quadruplex conformation and other higher-order structures. The presence of a mixture of monomeric and higher-order structures was confirmed using polyacrylamide gel electrophoresis. In order to further confirm the presence of higher-order structures, we pursued an approach where the formation of these structures would be prevented so as to achieve a monomer structure and then, using different techniques, use this “enforced” monomeric structure to compare with the higher-order structures naturally formed by the 11-mer. To achieve this, we investigated the extension of the 11-mer with further residues at the 3′ and/or 5′ end, an approach that has been reported to avoid the formation of higher-order structures and reduce G-quadruplex polymorphisms [29], and the addition of thymine residues at the sequence ends has been demonstrated to be particularly successful [31]. Three 11-mer-related sequences were, thus, designed in which a TT tail was added to the 3′ or/and the 5′ end of the original 11-mer sequence. The absence of broad signals in the range 10.5–11.0 ppm for the TT extension on the 5′ end (TT-11-mer and TT-11-mer-TT) suggests the prevention of the stacking effect. A similar effect was observed in the electrophoretic behavior of these TT-tethered 11-mers, where a single band was observed when TT extensions were added on the 5′ end, again demonstrating that this modification prevents the stacking effect, whilst the TT tail on the 3′ end showed bands attributable to both monomeric and dimeric forms, as the stacking effect was not prevented. This phenomena of preventing the stacking effect via extension of the 5′ end rather than the 3′ end can be attributed to a preference for 5-5 dimerization due to larger inter-phosphate distance provoking less electrostatic repulsion between the monomers [38]. MALDI-TOF also confirmed the influence of the TT extensions to prevent stacking when present at the 5′ end.

HPLC studies further highlighted the presence of two structures, monomeric and higher-order, as two distinct peaks were observed at different retention times. HLPC-ESI-TOF-MS clearly showed that the higher-order structure with the markedly longer retention time was a complex of the monomer.

CD suggests that the 11-mer adopts a major parallel G-quadruplex structure, although in co-existence with minor amounts of non-parallel conformation, and further highlighted that the presence of the TT at the 5′ end resulted in a monomer with parallel orientation. UV thermal melting analysis again demonstrated the difference in structures between the 11-mer and 11-mer-TT with respect to the TT-11-mer and TT-11mer-TT, with significantly higher concentration-dependent UV melting temperatures observed for the former, which can be attributed to the more complex higher-order structures present.

Biotinylated aptamers are commonly used in aptamer-based assays and sensors, and the effect of different types of spacers on the 11-mer structure was evaluated. The use of a nucleic acid poly-thymine spacer was compared with that of a carbon triethylene glycol spacer. In both cases, the formation of a parallel G-quadruplex structure was observed, which other techniques have suggested to be monomeric.

**Figure 8:**
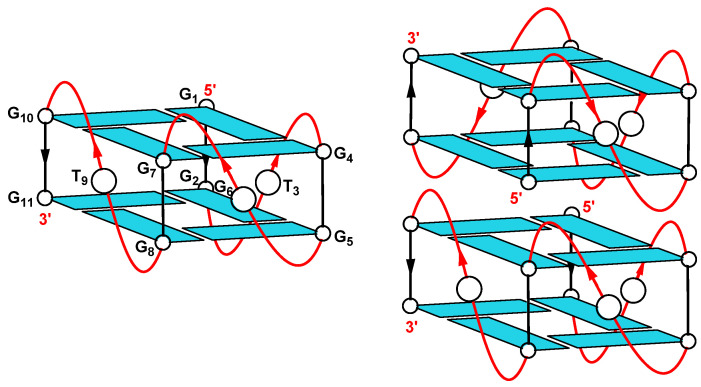
Schematic representation of the proposed parallel-stranded G-quadruplex structures, monomer (**left**) and head-to-head dimer (**right**), adopted by the 11-mer aptamer (5′-GGTGGGGGTGG-3′).

Based on these observations, we postulate a mixture of monomeric, dimeric and multiple order structures and Figure 8 shows a schematic representation of the proposed parallel-stranded G-quadruplex structures, monomer and dimer, adopted by the 11-mer aptamer.

Binding affinity studies revealed some interesting observations. Three techniques were used to evaluate the binding—BLI and MST, which was carried out independently by 2Bind GmBH, and SPR, which was carried out in house. The K_D_ elucidated for the 11-mer was similar using all three techniques and was in agreement with previous studies carried out using surface plasmon resonance imaging (SPRi) [39] and SPR [24]. Despite the clear difference between the higher-order structures observed with the 11-mer and the 11-mer-TT, and the monomeric structure obtained with the TT-11-mer and TT-11-mer-TT, the affinity constants were remarkably similar, all being in the nanomolar range.

## 4. Materials and Methods

### 4.1. Materials

All DNA sequences were purchased from Biomers.net (Germany) (Table S1), and β-conglutin was extracted from lupin seeds as previously described [40]. The aptamer binding buffer used for the assays was PBS (10 mM phosphate buffer, 135 mM NaCl, 2.7 mM KCl, pH 7.4) with 1.5 mM MgCl_2_.

### 4.2. Gel Electrophoresis

All oligonucleotides were analyzed by non-denaturing polyacrylamide gel electrophoresis (PAGE). Samples prepared in 10 mM KH_2_PO_4_, 40 mM KCl and 0.2 mM EDTA, pH 7 were loaded on a 20% polyacrylamide gel containing Tris–Borate-EDTA (TBE) 2.5× and 20 mM NaCl. The run buffer was 1× TBE containing 100 mM NaCl. For all samples, a solution of glycerol/(1× TBE and 50 mM NaCl) 2:1 was added just before loading. Electrophoresis was performed at 8 V/cm at a temperature close to 10 °C. Bands were visualized by UV shadowing.

### 4.3. Thermal Melting Analysis

UV thermal melting experiments were conducted using a VARIAN Cary 100 Bio spectrophotometer (Varian Iberica, Barcelona, Spain) with a temperature control accessory. Absorbance at 295 nm was recorded as a function of temperature ranging from 20 to 95 °C with heating and cooling rates of 1 °C/min. One hundred and twenty microliters of the 11-mer and its TT derivative oligonucleotides were heated to 95 °C for 5 min and then slowly cooled again prior to melting analysis. Experiments were performed with quartz cuvettes of 1 cm path length. The concentrations of the oligonucleotides studied ranged from 0.5 to 100 μM diluted in binding buffer with 20 mM KCl. In order to compare the results, a multicell thermal block was used, and samples were measured simultaneously.

### 4.4. Liquid Chromatography Coupled to Time-of-Flight Mass Spectrometry (HPLC-ESI-TOF-MS)

Samples were analyzed on a 1200 series HPLC coupled to a 6510 Time of Flight LC/MS (Agilent Technologies, Santa Clara, CA, USA), with an XDB-C18 column (4.6 mm × 50 mm, 1.8 μm, Agilent Technologies). An autosampler was used for injections of 10 μL. The column was equilibrated with 5 mM ammonium acetate pH 8 and eluted with a linear constant gradient during 15 min (from 5 to 100% of 50 mM ammonium acetate in 50% methanol pH 8) at a mobile phase flow of 0.4 mL/min. The absorbance of the fractions was measured at 260 nm. Mass spectrometer was operated on negative ionization mode using electrospray ionization. The drying gas temperature was set at 350 °C with a nitrogen flow rate of 9 L/min, the nebulizer pressure was 35 psi and capillary voltage was set at −2500 V. The mass spectrum was recorded in the range of 100–3000 m/z. The reference masses 1033,988109 and 980,016375 were used as internal calibration, and Mass Hunter Qualitative Analysis software was used for data processing.

### 4.5. Matrix-Assisted Laser Desorption/Ionization Time-of-Flight (MALDI-TOF-MS)

Analysis was performed by mixing the samples 1:1 with the matrix (3-hydroxypicolinic acid (3-HPA) and diammonium hydrogen citrate (DHAC), 9:1 *v*/*v*). The mixture was dropped onto an AnchorChip (Bruker Daltonik GmbH, Bremen, Germany) plate and analyzed with a MALDI-TOTF/TOF Ultraflex Extreme spectrometer (Bruker Daltonik) in a linear positive mode with a mass range between 1000–20,000 m/z.

### 4.6. Circular Dichroism (CD)

Each aptamer variant (100 μL of 10 μM) was prepared in binding buffer and annealed (heating for 5 min at 95 °C and slowly cooling to room temperature) prior to analysis. The CD spectra were acquired on a Chirascan CD spectrometer (Applied Photophysics, Leatherhead, UK) using a 10 mm path length quartz microcell in the range of 210–330 nm with a 1 nm step and adaptive sampling. Three scans were acquired for each sample, and after subtraction of the background (binding buffer), the scans were averaged and smoothed using the Pro-Data Chirascan software.

### 4.7. NMR

The NMR samples of the oligonucleotides were prepared at a concentration of about 1.6 mM, in 0.6 mL (H2O/D2O 9:1 *v*/*v*) buffer solution having 10 mM KH2PO4/K2HPO4, 40 mM KCl and 0.2 mM EDTA (pH 7.0). The samples were heated for 15 min at 95 °C and slowly cooled (10–12 h) to room temperature. The solutions were stored at 4 °C before analysis and then equilibrated at 25 °C for 2 h before performing experiments. The annealing process was assumed to be complete when 1H-NMR spectra were superimposable on changing time. NMR spectra were recorded with Varian Unity INOVA 500 MHz spectrometer (T = 25 °C). 1D proton spectra of the sample in H2O were recorded using pulsed-field gradient DPFGSE for H2O suppression. 1H-chemical shifts were referenced relative to external sodium 2,2-dimethyl-2-silapentane-5-sulfonate (DSS).

### 4.8. Surface Plasmon Resonance (SPR)

Surface plasmon resonance (SPR) was performed with BIAcore 3000 (Izasa SA, Barcelona, Spain). β-conglutin protein was immobilized on CM5 sensor chip activated with 3-(ethyliminomethyleneamino)-N, N-dimethylpropan-1-amine (EDC)/ N-hydroxysuccinimide (NHS) (30 µL of a 1:1 mixture of EDC (400 mM) and NHS (100 mM) followed by injection of 200 µg/mL protein at a flow rate of 5 µL/min). After immobilization of the protein, unreacted NHS esters were deactivated via injection of an excess of ethanolamine hydrochloride (1 M). Unbound protein was then washed from the surface with regeneration buffer (2 M NaCl, 10 mM NaOH) at 20 µL/min. The final immobilization level of β-conglutin was 6340 RU. The 11-mer aptamer and TT derivatives were diluted in binding buffer to the desired concentrations (up to 2.5 µM) and injected during 6 min at a flow rate of 10 µL/min followed by 3 min of stabilization time and 10 min of dissociation time. The binding of DNA was analyzed through the corresponding changes in the refractive index of the optical signal and expressed as resonance units (RU). The nonspecific binding was corrected by subtraction of an equal injection of the aptamers through an unmodified flow cell (without β-conglutin) blocked with ethanolamine and for zero concentration sample. The K_D_ was determined from the kinetic constants using a 1:1 binding model using the BioEvaluation software. Duplicates were carried out for all the experiments.

### 4.9. Biolayer Interferometry (BLI)

BLI studies were performed by 2Bind GmBH (http://2bind.de/molecularinteractions/) with an Octet K2 BLI instrument (Pall Forte Bio) based on biotin−streptavidin interactions. Biotinylated aptamer (biotin-TEG-T15-11mer) was immobilized on high-sensitivity streptavidin-activated biosensors (SAX) at a concentration of 1 μg/mL in binding buffer (Figure S6). Following immobilization, the sensors were blocked with biocytin (10 μg/mL). Serial dilutions of β-conglutin ranging from 100 to 1.2 nM were prepared in assay buffer (PBS, 1.5 mM MgCl_2_, pH 7.4, 0.1% Tween-20). The samples were analyzed at 30 °C. Regeneration buffer (2 M NaCl, 10 mM NaOH) was used to wash the biosensor between measurements. As a control reference, a sample without β-conglutin was used (Figure S6). The binding was analyzed through the signal shift in nanometers, and data were fitted globally to a 2:1 heterogeneous binding model. Duplicates were carried out for all the experiments.

### 4.10. MicroScale Thermophoresis (MST)

MST binding experiments were carried out with 1 nM Cy5-labeled 11-mer aptamer and TT derivatives in binding buffer with 0.05% *v*/*v* Tween-20 with varied concentrations of β-conglutin at 40% MST power, 20% LED power in standard capillaries on a Monolith NT.115 pico device at 25 °C (NanoTemper Technologies, Munich, Germany). The reaction buffer used to characterize the 11-mer derivative sequences (TT-11-mer-TT, TT-11-mer and 11-mer-TT) contained 20 mM KCl. To analyze the MST data, the recorded fluorescence was normalized to fraction bound (0 = unbound, 1 = bound), processed using the software KaleidaGraph 4.5 and fitted using the EC50 fitting formula from the Hill equation. Duplicates were performed for each experimental setup.

## 5. Conclusions

The structural forms of the shortest known G-quadruplex-containing aptamer was investigated using a battery of techniques, all of which corroborated the co-existence of monomeric and multimeric higher-order structures. Initially, gel electrophoresis showed the existence of multiple forms, which was confirmed by HPLC and HPLC combined with MALDI-TOF-MS, which also clearly show the existence of both monomeric and multimeric structures. Thermal melting analysis, as well as NMR and CD, further confirm these multiple structures. The addition of a TT-tail at the 5′ end prevented the stacking effect and effectively hindered the formation of higher-order structures. In the biotinylated form of the aptamer, a TEG carbon prevented it from folding in more complex structures. Interestingly, independent of the structural forms present, the 11-mer truncated aptamer was observed to have nanomolar affinity. The work carried out can conclude that the 11-mer, the shortest known, G-quadruplex-containing, aptamer, can exist in monomeric, dimeric and multimeric forms but, as is clearly indicated by the MST and SPR studies, irrespective of the structure, maintains its affinity with its cognate target β-conglutin.

## Data Availability

The data presented in this study are available on request from the corresponding author.

## Supplementary Materials

Supplementary materials can be found at https://www.mdpi.com/1422-0067/22/3/1150/s1. Table S1. Oligonucleotide sequences used in this work. Table S2. UV melting temperatures of the 11-mer and its TT derivatives. Figure S1. MALDI-TOF analysis of the 11-mer and its TT derivatives.

## Abbreviations

BLI	Biolayer interferometry
CD	Circular dichroism
EDC	3-(Ethyliminomethyleneamino)-N, N-dimethylpropan-1-amine
EDTA	Ethylenediaminetetraacetic acid
ESI-TOF-MS	Electrospray ionization time-of-flight mass spectrometry
FRET	Fluorescence resonance energy transfer
HPLC	High-performance liquid chromatography
K_D_	Affinity dissociation constant
MALDI	Matrix-assisted laser desorption/ionization
MST	Microscale thermophoresis
NHS	N-Hydroxysuccinimide
NMR	Nuclear magnetic resonance
PAGE	Polyacrylamide gel electrophoresis
SAX	High-sensitivity streptavidin-activated biosensors
SELEX	Systematic Evolution of Ligands by Exponential Enrichment
SPR	Surface plasmon resonance
SPRi	Surface plasmon resonance imaging
T15	15× thymine spacer
TBA	Thrombin binding aptamer
TBE	Tris-borate/EDTA
TEG	Triethylene glycol
UV	Ultraviolet
