# Supplementary material for: Aptamers Against the β-Conglutin Allergen: Insights into the Behavior of the Shortest Multimeric(Intra)Molecular DNA G-Quadruplex
Source: Int J Mol Sci. 2021 Jan 24;22(3):1150. doi: 10.3390/ijms22031150 (PMC7865891; doi:10.3390/ijms22031150)
Supplement: Supplementary file 1 [file ijms-22-01150-s001.pdf]

Supplementary Materials

Aptamers Against the β-Conglutin Allergen: Insights into the Behaviour of the Shortest Multimeric (Intra)Molecular DNA G-Quadruplex

Ciara O'Sullivan [1,2,*], Teresa Mairal [1,†], Miriam Jauset-Rubio [1,†], Marketa Svobodova [1,†], Vasso Skouridou [1,†,] Veronica Esposito [3], Antonella Virgilio [3] and Aldo Galeone [3]

1 INTERFIBIO Research Group, Departament d'Enginyeria Química, Universitat Rovira i Virgili, Avinguda Països Catalans 26, 43007 Tarragona, Spain; teresa.mairal@urv.cat (T.M.); miriam.jauset@urv.cat (M.J.-R.); marketa.svobodova@urv.cat (M.S.); vasoula.skouridou@urv.cat (V.S.)
2 Institució Catalana de Recerca i Estudis Avançats (ICREA), Passeig Lluís Companys 23, 08010 Barcelona, Spain
3 Dipartimento di Farmacia, Università degli Studi di Napoli Federico II, Via D. Montesano 49, 80131 Napoli, Italy; verespos@unina.it (V.E.); antonella.virgilio@unina.it (A.V.); galeone@unina.it (A.G.)
* Correspondence: ciara.osullivan@urv.cat
† These authors have contributed equally to the work.

<u>Table of contents:</u>

Table S1. Sequences used in this work. 2

Table S2. UV melting temperatures of 11-mer and TT derivatives when used at 100 μM each..... 2

Figure S1. MALDI-TOF analysis of the 11-mer and TT derivatives. 3

Figure S2. UV melting curves of the 11-mer and TT derivatives when used at 100 μM each. 4

Figure S3. CD spectra of the 11-mer and derivative sequences. (a) 11-mer and TT derivatives (10 μM each) were analysed in PBS with 1.5 mM $MgCl_2$ and 2.7 mM KCl at 25 °C. (b) 11-mer and biotinylated derivatives (10 μM each) were analysed at different concentrations of KCl, 2.7 mM and 20 mM, in PBS with 1.5 mM $MgCl_2$ at 25°C. 5

Figure S4. BLI binding studies for the 11-mer aptamer with β-conglutin. Fitting of the curves was performed using (a) a 1:1 binding model and (b) a 2:1 heterogeneous model.......... 6

Figure S5. Target immobilization for BLI binding studies. (1) Initial baseline prior to target immobilization; (2) loading of the biotinylated target; (3) blocking of the streptavidin biosensor with biocytin; (4, 6 and 8) equilibration; (5, 7 and 9) regeneration. 6

Figure S6. BLI sensograms obtained for the 11-mer aptamer after the addition of different concentrations of β-conglutin. 7

Table S1. Sequences used in this work.

Name	Sequence (5′ - 3′)	Length	Modifications
11-mer	ggtgggggtgg	11	
TT-11-mer	tt-ggtgggggtgg	13	
11-mer-TT	ggtgggggtgg-tt	13	
TT-11-mer-TT	tt-ggtgggggtgg-tt	15	
Cy5-11-mer	ggtgggggtgg	11	5´Cy5
Cy5-TT-11-mer	tt-ggtgggggtgg	13	5´Cy5
Cy5-11-mer-TT	ggtgggggtgg-tt	13	5´Cy5
Cy5-TT-11-mer-TT	tt-ggtgggggtgg-tt	15	5´Cy5
Biotin-TEG-T15-11-mer	ttttttttttttttt-ggtgggggtgg	26	5´ Biotin-TEG-T15
TBA	ggttggtgtggttgg	15	

Table S2. UV melting temperatures of 11-mer and TT derivatives when used at 100 μM each.

Sequence	Tm (°C)
11-mer	76
TT-11-mer	61
11-mer-TT	75
TT-11-mer-TT	62

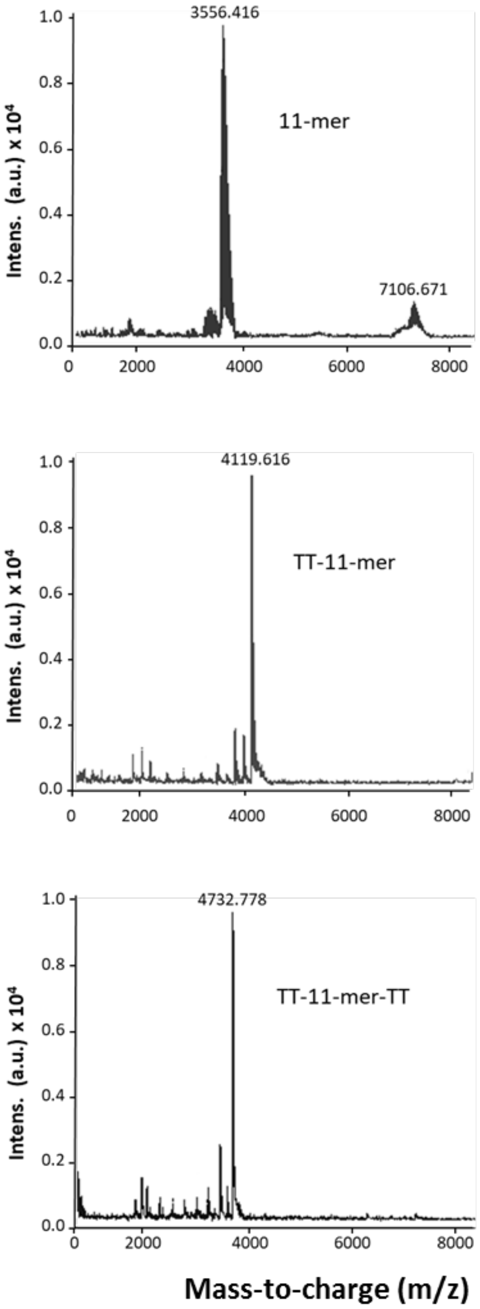

Figure S1. MALDI-TOF analysis of the 11-mer and TT derivatives.

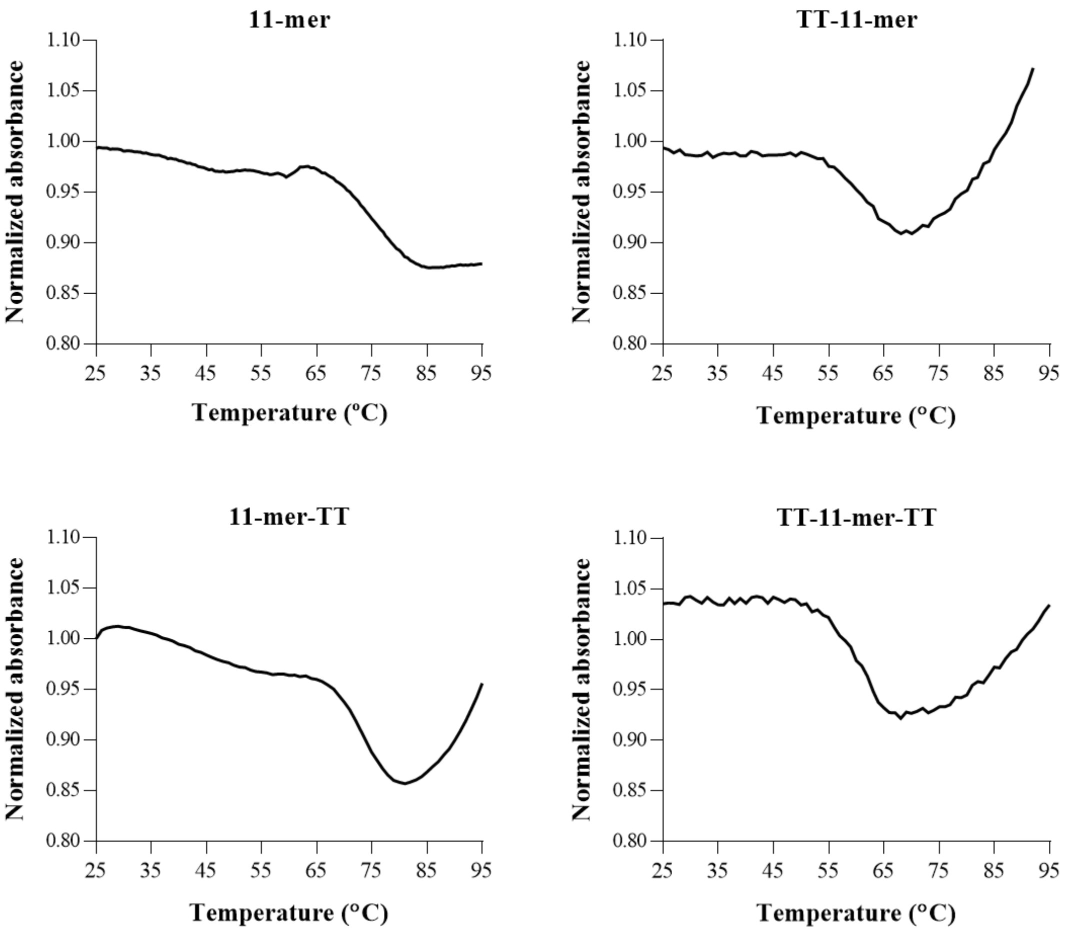

Figure S2. UV melting curves of the 11-mer and TT derivatives when used at 100 μM each.

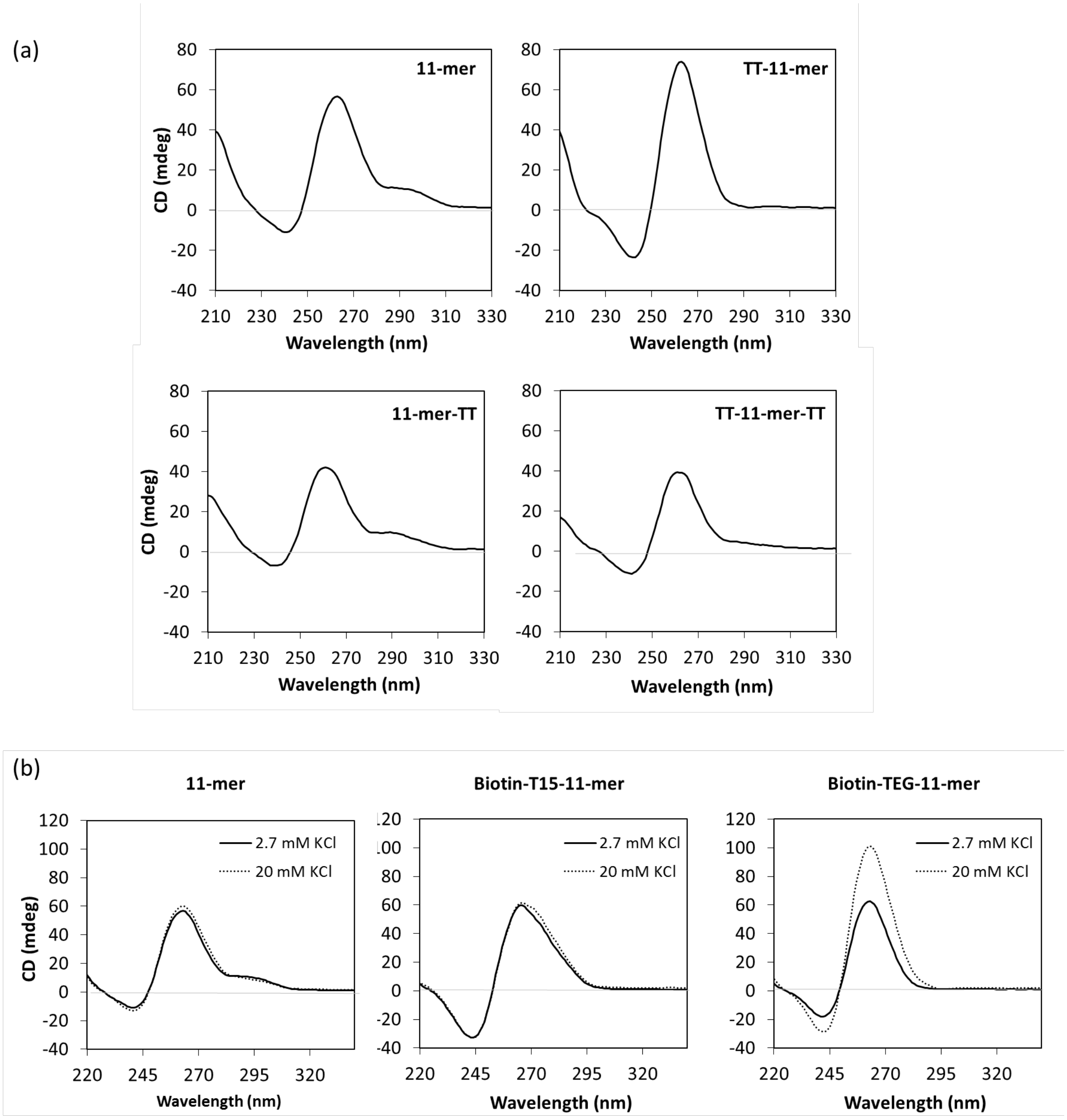

Figure S3. CD spectra of the 11-mer and derivative sequences. (a) 11-mer and TT derivatives (10 μM each) were analysed in PBS with 1.5 mM $MgCl_2$ and 2.7 mM KCl at 25 °C. (b) 11-mer and biotinylated derivatives (10 μM each) were analysed at different concentrations of KCl, 2.7 mM and 20 mM, in PBS with 1.5 mM $MgCl_2$ at 25°C.

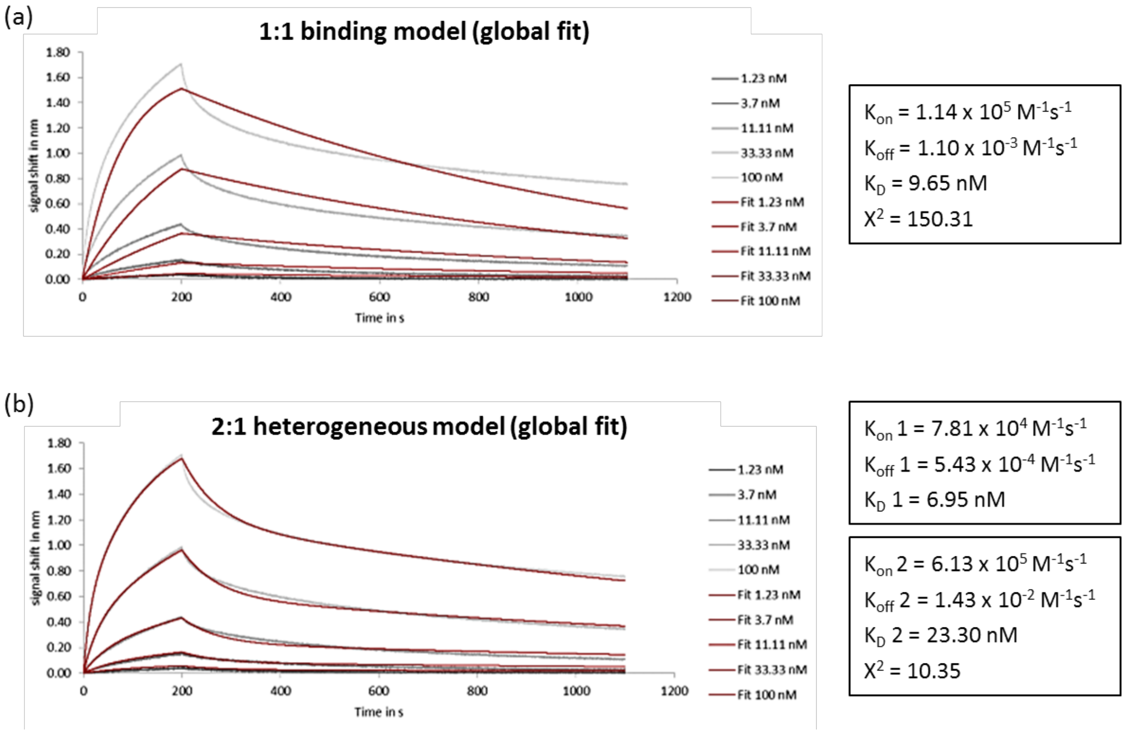

Figure S4. BLI binding studies for the 11-mer aptamer with β-conglutin. Fitting of the curves was performed using (a) a 1:1 binding model and (b) a 2:1 heterogeneous model.

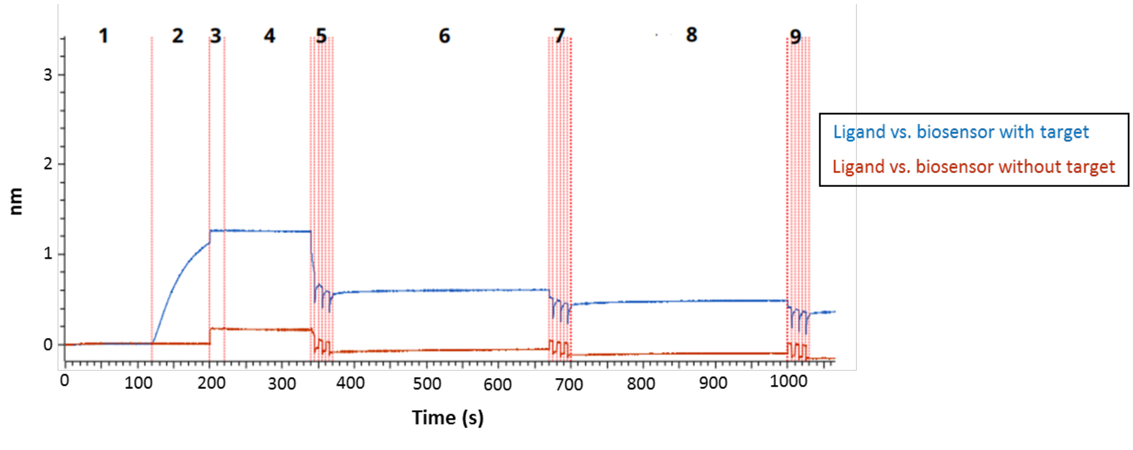

Figure S5. Target immobilization for BLI binding studies. (1) Initial baseline prior to target immobilization; (2) loading of the biotinylated target; (3) blocking of the streptavidin biosensor with biocytin; (4, 6 and 8) equilibration; (5, 7 and 9) regeneration.

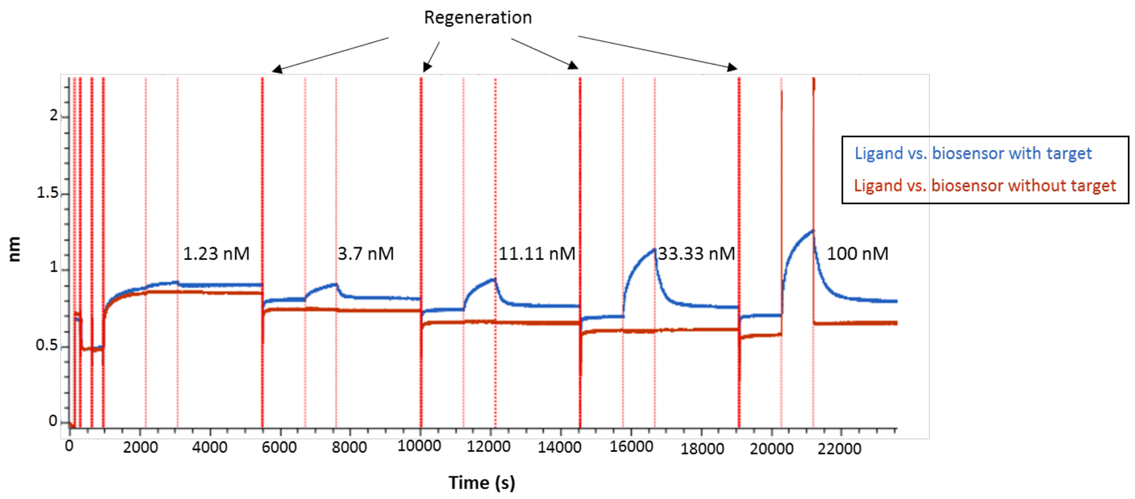

Figure S6. BLI sensograms obtained for the 11-mer aptamer after the addition of different concentrations of β-conglutin.
